# A Case of Transient ST Elevation and Polymorphic Tachycardia without Angina Diagnosed by Holter Monitoring

**DOI:** 10.7759/cureus.1273

**Published:** 2017-05-25

**Authors:** Ali Farooq, Fahad Alqahtani, Almoutassim Trabulsi, Akram Kawsara, Mohamad Alkhouli

**Affiliations:** 1 Internal Medicine, West Virginia University - Charleston Division; 2 Division of Cardiovascular Disease, West Virginia University School of Medicine/Ruby Memorial Hospital

**Keywords:** polymorphic ventricular tachycardia, holter monitor, silent myocardial infarction

## Abstract

We report the case of 52-year-old female with recurrent episodes of palpitations and dizziness. Holter monitoring revealed transient ST elevations followed by episodes of polymorphic ventricular tachycardia associated with episodes of palpitations and dizziness. Coronary angiography revealed mildly irregular right coronary artery with 90% stenosis. The patient underwent percutaneous coronary intervention with successful placement of a stent to the mid-right coronary artery. The patient has been followed closely over a period of 12 months. There haven't been any recorded episodes of tachycardia, and the patient has remained symptom-free.

## Introduction

The diagnosis of acute myocardial infarction is mainly based on the electrocardiogram (EKG) findings of ST elevations or new onset left bundle branch block, which is supported by the clinical presentation and positive biomarkers when present. Although anginal episodes (chest pain, dyspnea, and diaphoresis) are the cardinal symptoms of acute myocardial, silent myocardial infarction (SMI) occurs far more frequently than angina episodes in patients with stable coronary artery disease. In a study of 105 patients with stable angina, 40% of patients have frequent episodes of SMI on ambulatory EKG monitoring [1]. We report a case of transient ST-elevation and polymorphic ventricular tachycardia without angina diagnosed by Holter monitoring.

## Case presentation

**Figure 1:**
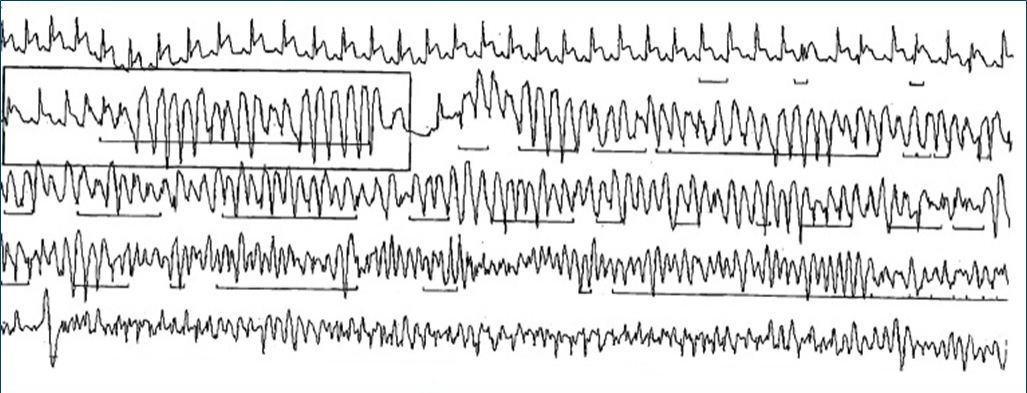
Fragment from 24-hour Holter monitor showing ST elevation followed by polymorphic ventricular tachycardia.

**Figure 2:**
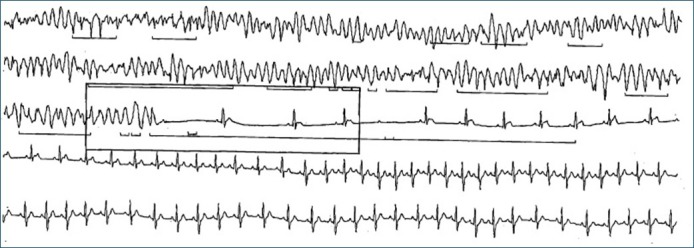
Fragment from 24-hour Holter monitor showing polymorphic ventricular tachycardia and Mobitz Type II atrioventricular block followed by sinus tachycardia.

A 52-year-old female with a history of hypertension, hyperlipidemia, and Raynaud's syndrome but no prior history of coronary artery disease presented with recurrent episodes of palpitations and dizziness. Clinical examination and initial investigations were normal. The initial 12-lead ECG showed sinus rhythm. Echocardiogram, chest X-ray, routine biochemical, troponins, and hematological analyses were normal. A Holter monitor obtained for further evaluation revealed transient ST elevations followed by significant episodes of polymorphic ventricular tachycardia (Figure 1) and Mobitz Type II atrioventricular block (Figure 2).

**Figure 3:**
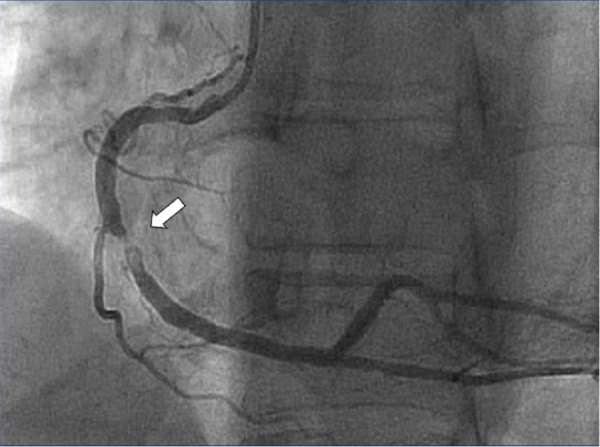
Coronary angiography demonstrating mild right coronary artery irregularity with a 90% focal lesion with thrombus.

Surprisingly, no complaint of angina was reported during the period of monitoring. The palpitations and dizziness corresponded to episodes of fast polymorphic ventricular tachycardia. The patient was admitted for further evaluation. Repeated EKG showed nonspecific changes in the inferior leads. Cardiac biomarkers and electrolytes were within normal limits. Coronary angiography demonstrated a mild right coronary artery irregularity with a 90% focal lesion with thrombus (Figure 3).

She underwent percutaneous coronary intervention with the successful placement of a stent to the mid-right coronary artery as well as aspiration thrombectomy. The patient has been followed closely over a period of 12 months. There haven't been any recorded episodes of tachycardia and patient has remained symptom-free.

## Discussion

It is well described that a certain group of patients does not display the typical symptoms of myocardial infarction. Elderly patients, diabetics, and those with previous coronary artery bypass graft surgery are at high risk for SMI [2]. The diagnoses can sometimes become challenging when the patient is asymptomatic or presents with atypical symptoms [3-4]. Ambulatory EKG monitoring can be used as a diagnostic test for detecting SMI in high-risk patients. It has improved the likelihood of recording ischemic and arrhythmic episodes during routine daily activities out of the hospital setting.

Some previous reports showed Holter recordings of ST-segment elevations and ventricular arrhythmias secondary to vasospastic angina. A case of sudden death secondary to vasospastic angina during Holter monitoring has been reported; ventricular fibrillation occurred after several ventricular premature beats following ST-segment elevation [5]. Another similar case of polymorphic ventricular tachycardia degenerating in ventricular fibrillation has been reported that occurred during Holter recording in a patient with recurrent vasospastic angina and normal coronary arteries [6].

A high index of suspicion is needed, especially in high-risk patients. SMI, when detected during ambulatory monitoring, predicts advanced multivessel coronary artery disease. In a study of 439 patients with SMI, 75% of patients were found to have multivessel coronary artery disease on coronary angiography [7]. There is evidence in the literature showing a strong association of SMI with increased cardiac mortality [8].

## Conclusions

Our case endorses that SMI can be observed in patients without a history of known coronary artery disease. We think that in the rare clinical situation when the diagnosis is in doubt, SMI should be ruled out by Holter monitoring since SMI has a strong association with cardiac mortality. Early detection and initiation of therapy with medical management or revascularization in appropriate settings may improve prognosis in this patient population.
